# Bispecific T-Cell Engagers Targeting Membrane-Bound IgE

**DOI:** 10.3390/biomedicines9111568

**Published:** 2021-10-29

**Authors:** Aleksandra Rodak, Gerhard Stadlmayr, Katharina Stadlbauer, Dominic Lichtscheidl, Madhusudhan Reddy Bobbili, Florian Rüker, Gordana Wozniak-Knopp

**Affiliations:** 1 Institute of Molecular Biotechnology, Department of Biotechnology, University of Natural Resources and Life Sciences, Vienna (BOKU), Muthgasse 18, 1190 Vienna, Austria; aleksandra.rodak@boku.ac.at (A.R.); gerhard.stadlmayr@boku.ac.at (G.S.); katharina.stadlbauer@boku.ac.at (K.S.); dominic.lichtscheidl@gmail.com (D.L.); madhusudhan.bobbili@boku.ac.at (M.R.B.); florian.rueker@boku.ac.at (F.R.); 2 Ludwig Boltzmann Institute for Experimental, Clinical Traumatology in the AUVA Research Center, Donaueschingenstrasse 13, 1200 Vienna, Austria

**Keywords:** anti-IgE antibodies, bispecific T-cell engagers, cytotoxic T-lymphocyte mediated killing, extracellular membrane-proximal domain, Fcε, T-cell activation

## Abstract

The increased incidence of allergies and asthma has sparked interest in IgE, the central player in the allergic response. Interaction with its high-affinity receptor FcεRI leads to sensitization and allergen presentation, extracellular membrane-proximal domain in membrane IgE can act as an antigen receptor on B cells, and the interaction with low-affinity IgE receptor CD23 additionally influences its homeostatic range. Therapeutic anti-IgE antibodies act by the inhibition of IgE functions by interfering with its receptor binding or by the obliteration of IgE-B cells, causing a reduction of serum IgE levels. Fusion proteins of antibody fragments that can act as bispecific T-cell engagers have proven very potent in eliciting cytotoxic T-lymphocyte-mediated killing. We have tested five anti-IgE Fc antibodies, recognizing different epitopes on the membrane-expressed IgE, for the ability to elicit specific T-cell activation when expressed as single-chain Fv fragments fused with anti-CD3ε single-chain antibody. All candidates could specifically stain the cell line, expressing the membrane-bound IgE-Fc and bind to CD3-positive Jurkat cells, and the specific activation of engineered CD3-overexpressing Jurkat cells and non-stimulated CD8-positive cells was demonstrated for 8D6- and ligelizumab-based bispecific antibodies. Thus, such anti-IgE antibodies have the potential to be developed into agents that reduce the serum IgE concentration by lowering the numbers of IgE-secreting cells.

## 1. Introduction

In the past decade, IgE antibodies have risen to the limelight of the antibody research community, on the one hand, due to their unique mode of target engagement via variable regions [1], and on the other hand, due to the extreme conformational changes these molecules undergo upon binding with their receptors; this flexibility is a prerequisite for their activity and can be modulated for therapeutic purposes [2]. The IgE antibody class, with very low concentrations, of about 150 ng/L in plasma, compared with the most abundant IgG, of about 10 mg/L [3], is critically involved in mediating allergic reactions through the vigorous activation of the effector functions mediated by binding to Fc receptors FcεRI and FcεRII/CD23 [4]. One of the first concepts employed for the anti-IgE therapy was the inhibition of the interaction of Fcε with its cognate receptors, either by steric or allosteric means [5]. As a prominent example, the treatment of uncontrolled allergic asthma demonstrated the effectiveness of anti-IgE-specific antibody omalizumab (Xolair^®^) [4,5]. Utilizing its high antigen affinity, at least an order of magnitude higher than that of common therapeutics of the IgG class, omalizumab prevents IgE binding to FcεRI and CD23 and rapidly reduces the serum concentration of IgE [6]. At the same time, the research into the anti-IgE therapies provides evidence that IgE-directed antibody-based approaches could benefit from targeting B lymphocytes displaying membrane-bound IgE, adding to rapid reduction of soluble IgE by the obliteration of IgE-secreting plasma cells. The first attempts to eliminate IgE-B cells employed genetically modified T cells carrying a chimeric anti-IgE T-cell receptor [7]. Furthermore, anti-IgE antibodies, formed as a part of an individual’s immune response, could inhibit the IgE synthesis in vivo and provide tolerogenic signals for IgE memory B cells over the interaction with membrane-bound IgE [8]. Even passive immunization with antibodies targeting the extracellular membrane–proximal domain (EMPD) of IgE suppressed an IgE response to the simultaneously introduced birch pollen allergen in mice [9]. Recent reports describe IgE-specific cytotoxic T lymphocytes (CTLs) generated ex vivo, which can effectively lyse IgE-producing B cells in vitro and CTLs that, after adoptive transfer, downregulate IgE responses and ameliorate airway inflammation in an asthmatic mouse model [10].

IgE-Fc targeting antibodies, many of which have entered the stage of clinical testing, target a wide range of epitopes, resulting in very diverse profiles of their activities and differing modes of action [2]. Omalizumab binds to a partially bent conformation of the Cε3 domains and can also achieve 2:1 stoichiometry; upon binding, Cε3 domains adopt a very “open” conformation [11]. Another anti-IgE antibody, 8D6, recognizes Cε2 and Cε3 domains in an extended conformation; in the complex, the Cε2 domain pair is pressed towards the Cε3 domains [12]. This antibody does not recognize the IgE bound by FcԑRI; it can, however, in contrast to omalizumab, also target CD23-bound IgE [13]. Apart from being able to neutralize IgE without concomitant activation of mast cells and basophils, it can crosslink CD23 on B cells and thereby inhibit the synthesis of IgE.

Ligelizumab is a humanized IgG1 anti-IgE antibody progressing in clinical development [14] that binds to the Cε3 domain with a higher affinity than omalizumab. Although the epitopes of the two antibodies significantly overlap, the recognition regions clearly differ [15]. Due to a different angle of binding of the two antibodies, their receptor inhibition functions are distinct, and recent reports suggest that the efficiency of ligelizumab results from its more potent blocking of the FcԑRI than CD23 interaction [15].

Another antibody, MEDI4212, has entered clinical trials and could neutralize soluble IgE more rapidly than omalizumab [16], by binding selectively to the Cε3 and Cε4 domains and inhibiting the interaction of IgE with FcεRI and CD23. Its affinity for IgE is 1.95 pM in vitro, about one hundred times higher than that of omalizumab [17]. MEDI4212 can inhibit calcium signaling in mast cells with 30–100-fold the potency of omalizumab, also effectively reducing the IgE responses through CD23, and its variants with enhanced affinity for FcγRIII have the potential to eliminate the IgE-expressing B cells efficiently before their conversion to IgE-secreting plasma cells [18].

Quilizumab targets the CεmX-containing fragment of cell-expressed IgE, positioned between the C_H_4 domain of IgE and its B-cell membrane-anchoring segment [19] and does not react with soluble IgE. By crosslinking of the membrane-bound IgE antigen receptors on B cells, IgE+B cell apoptosis is induced, and thereby inhibits the generation of IgE+B cells and efficiently reduces soluble IgE serum levels. Additionally, it can deplete IgE-switched and memory B cells through antibody-dependent cell-mediated cytotoxicity (ADCC) [20].

A very efficient mode of elimination of specific cell groups has been demonstrated for a class of bispecific antibodies, which can simultaneously specifically engage target cells and activate T-mediated cell killing by CTL [21]. Certain limitations of other bispecific antibody formats that could act in this way have been overcome with the bispecific T-cell engagers (BiTEs), which consist of minimal antigen-binding domains of two different monoclonal antibodies in a single polypeptide chain [22]. Such therapies have been introduced into clinical practice with blinatumomab (Blincyto™), approved for the treatment of acute lymphocytic leukemia in 2014 [23]. This molecule, composed of two single-chain antibodies connected with a short linker, binds to CD19, which is strongly overexpressed on the surface of B-cells, and to the ε-subunit of the CD3 on T-lymphocytes. The potent activity of blinatumomab has been led back to the ability of CTL expansion independently of co-stimulation and mediation of serial killing at low effector to target cell ratios [24]. Indeed, the induction of specific cytotoxicity against target cells expressing human transmembrane IgE by a bispecific anti-IgE/anti-CD3 molecule has already been described [25]. We were interested in examining the extent of T-cell activation that can be achieved by bispecific single-chain antibodies targeting various anti-IgE epitopes located in the membrane-bound IgE-Fc. For this purpose, variable domains of omalizumab, 8D6, ligelizumab, MEDI4212 and quilizumab were expressed in a BiTE-like format, tested for binding to cell-bound IgE-Fc and CD3, and for the ability to elicit specific T-cell activation.

## 2. Materials and Methods

### 2.1. Construction of Bispecific Anti-IgE Antibodies

Variable sequences of the anti-IgE antibodies were ordered as DNA strings from Geneart (Thermo Fisher Scientific, Waltham, MA, USA) (sequences in Supplementary Table S1) and amplified with PCR using primers listed in Supplementary Table S2. The sequence of blinatumomab was cloned to be used as a positive control for cell staining and T-cell activation experiments and cloned in frame with a C-terminal 8-His-tag, into the pTT28 vector (Canadian National Research Council, Ottawa, ON, Canada) after digestion with the corresponding restriction enzymes (New England Biolabs, Ipswich, MA, USA). Fragments encoding a single-chain fragment of the variable domains of anti-IgE antibodies were cloned into the existing construct to replace CD19-targeting domains.

### 2.2. Expression and Purification of Single-Chain Constructs

ExpiCHO cells (Thermo Fisher Scientific, Waltham, MA, USA) were cultured at 37 °C and 8% CO_2_ in a hydrated atmosphere on an orbital shaker at 125 rpm. Plasmid DNA was isolated from *E. coli* TOP10 (Thermo Fisher Scientific, Waltham, MA, USA) using midipreparation (Macherey-Nagel, Düren, Germany), sterilized with Ultrafree-MC centrifugal filter units (Merck Millipore, Burlington, MA, USA), and used for transfection as recommended by the manufacturer. Briefly, cells were transfected at a density of 5 × 10^6^/mL with 0.8 µg DNA per mL culture using Expifectamine. On the next day, Enhancer and ExpiCHO Feed were added, the incubation temperature was decreased to 28 °C and the CO_2_ concentration to 5%. Cultivation proceeded for 14 days with the addition of ExpiCHO Feed on day 5 post-transfection. Immobilized metal affinity chromatography (IMAC) was used to isolate the bispecific constructs. Supernatants of the expressing cultures were clarified by a centrifugation step at 2000× *g*, 15 min at 4 °C and filtration through a 0.45-µm filter. The samples were buffered with phosphate-buffered saline (PBS), pH 7.5, and loaded onto a HisTrap Excel chromatography column (Cytiva, Marlborough, MA, USA) equilibrated with the same buffer. After washing with PBS, weakly bound proteins were removed from the column with PBS/20 mM imidazole, pH 7.5. Fractions containing the bispecific protein were eluted using a gradient from 20–500 mM imidazole in PBS, pH 7.5, in 5 column volumes. The eluted fractions were inspected on a Coomassie-stained sodium dodecylsulphate-polyacrylamide gel electrophoresis (SDS-PAGE) gel, pooled, and dialyzed against PBS, pH 7.5, for 48 h at 4 °C. The protein concentration was determined using the measurement of A_280_, and the preparations were stored at 4 °C until further use.

### 2.3. High-Pressure Liquid Chromatography (HPLC)-Size Exclusion Chromatography (SEC) Analysis

A Shimadzu (Kyoto, Japan) LC-20A Prominence system equipped with a diode array detector was used to perform HPLC-SEC with a *Superdex* 200 Increase 10/300 GL column (Cytiva, Marlborough, MA, USA) in PBS with 200 mM NaCl as the mobile phase buffer. A total of 20 µg of protein at about 1 mg/mL was loaded on the column and eluted at a constant flow rate of 0.75 mL/min. Column calibration was performed with a set of molecular weight standards ranging from 670 to 1.3 kDa (Bio-RAD, Hercules, CA, USA).

### 2.4. SDS-PAGE

A total of 2 µg of purified protein preparations were mixed with loading sample buffer and resolved on 4–12% Novex NuPAGE gels, run in MES buffer for 35 min at 200 V, stained with a NovexBlue staining kit (all chemicals from Thermo Fisher Scientific, Waltham, MA, USA), and destained overnight with distilled water.

### 2.5. Cell Culture

An EMPD expressing cell line (Ramos EHRB) cell line stably transformed with all-in-One TET-inducible lentiviral HIV-based construct encoding IgE-Fc-B-cell receptor (BCR) encompassing 3xFLAG-Cε2-Cε3-Cε4-EMPD-transmembrane (TM)-intracellular domain (IC) [26] (kind gift of Oskar Smrzka and Günther Staffler, Affiris AG, Vienna, Austria) and Ramos EHRB cells transformed with an empty vector were cultivated in RPMI-1640 with 2 mM L-glutamine, sodium pyruvate, 100 U/mL penicillin, and 100 μg/mL streptomycin with 0.3 µg/mL G-418 (all from Thermo Fisher Scientific, Waltham, MA, USA) and 10% fetal calf serum (FCS) (Sigma-Aldrich, St. Louis, MO, USA), at 37 °C under 5% CO_2_ in a hydrated atmosphere. Cell surface expression of IgE BCR was monitored as a function of inductor doxycycline (Clontech, Takara Bio, Kusatsu, Gumma, Japan) concentration over a period of 48 h by staining with the control anti-IgE antibody omalizumab (Roche, Basel, Switzerland) and set at 1 µg/mL as optimum. CD3-positive Jurkat T-cell line, Clone E6-1 (ATCC^®^ TIB-152^™^), and CD3-negative T cells J.RT3-T3.5 (ATCC^®^ TIB-153^™^) were obtained from ATCC (Manassas, VA, USA) and cultured in the same medium without the addition of G-418.

### 2.6. Cell Surface Staining

Cell count and viability determination was performed with the Trypan-blue exclusion method with TC20 Automated Cell Counter (Bio-RAD, Hercules, CA, USA). Cells were harvested with centrifugation at 300× *g* for 5 min at 4 °C, resuspended in 2% ice-cold bovine serum albumin (BSA-PBS) at a density of 2 × 10^6^ cells/mL, blocked for 30 min on ice, and distributed into the wells of a 96-U-shaped-well plate in 100 µL-aliquots. All stainings were performed in duplicates. After centrifugation at 300× *g* for 5 min at 4 °C, the blocking solution was removed, and the pellets were resuspended in 100 µL of graded concentrations of bispecific antibodies in 2% BSA-PBS. After a 30-min-incubation on ice, the cells were collected with another centrifugation step and incubated in 100 µL/well of anti-pentahis-AlexaFluor^®^ 488 conjugate (QIAgen, Hilden, Germany) for the staining of Ramos cells, and anti-pentahis-AlexaFluor^®^ 647 conjugate (QIAgen, Hilden, Germany) for Jurkat and TIB-153 cells, diluted to 0.25 µg/mL in 2% BSA-PBS. After a final centrifugation step, the cells were resuspended in 200 µL ice-cold PBS, and 10,000 cells per sample were analyzed with a Guava^®^ EasyCyte™ Flow Cytometer (Luminex, Austin, TA, USA). The data on sample fluorescence were processed using Kaluza software version 2.1 (Beckman Coulter, Brea, CA, USA) to obtain the geometric mean of cell fluorescence, measuring the signal from bound anti-pentahis-AlexaFluor^®^ 488 or 647 conjugate and EC_50_ of cell surface binding was determined using Prism software version 5.03 (GraphPad Software, San Diego, CA, USA). Bispecific antibody samples of different batches were used in two independent experiments, and all stainings were performed at least in duplicates.

For monitoring of the Fcε expression of the transformed cell line, omalizumab was used in a concentration range from 10–0.01 nM. The Ramos EHRB cell line expressing the Fcε (Ramos-Fcε) and Ramos EHRB transformed with an empty vector were induced for 48 h with 1 µg/mL doxycycline, and not-induced Ramos-Fcε cells were included as a control. After 30-min-incubation on ice, the cells were collected with centrifugation, resuspended in 100 µL of anti-human-IgG-phycoerythrin (PE) conjugate (Sigma-Aldrich, St. Louis, MO, USA), diluted 1:800 in 2% BSA-PBS, and incubated for 30 min on ice. A final centrifugation step was followed by resuspending the cells in 200 µL of ice-cold PBS. Samples were analyzed in duplicates. FACS analysis and data evaluation were performed as described above by determining the geometric mean of cell fluorescence in FL2, measuring the signal from bound anti-human-IgG-phycoerythrin (PE) conjugate. The binding of quilizumab was tested with the same method, using the antibody in a concentration range from 100–0.1 nM.

### 2.7. Immunofluorescence Microscopy

The cells were harvested with centrifugation at 300× *g* for 5 min at 4 °C, resuspended in 2% (BSA-PBS) at a density of 1 × 10^6^ cells/mL, blocked for 30 min on ice, and distributed into the wells of a 96-U-shaped-well plate in 100 µL-aliquots. All stainings were performed in duplicates. After centrifugation at 300× *g* for 5 min at 4 °C, the blocking solution was removed. Ramos EHRB-Fcε cells and Ramos-EHRB cells transformed with the empty vector were resuspended in 100 µL of 100 nM bispecific antibody in 2% BSA-PBS, and for the staining of Jurkat and TIB-153 cells, the 60 nM concentration was used. After a 30-min-incubation on ice, the cells were collected with another centrifugation step and incubated in 100 µL/well of anti-pentahis-AlexaFluor^®^ 647 conjugate (QIAgen, Hilden, Germany), diluted to 0.2 µg/mL in 2% BSA-PBS. The cells stained with the secondary reagent served as a control. After a final centrifugation step, the cells were resuspended in 400 µL ice-cold PBS and 10 µL were delivered into a well of a 4-well-microinsert in a 35 µM µ-dish (ibidi, Gräfelfing, Germany). Samples were analyzed with a Leica DMI6000B microscope (Leica Microsystems, Wetzlar, Germany) using HCX Objective Plan-Apochromat 63x/1.4 Oil. Data on sample fluorescence were processed using Leica Application Suite X software, version 3.7.0 (Leica Microsystems, Wetzlar, Germany).

### 2.8. Internalization of Fcε

Induced Ramos-Fcε cells were harvested and incubated with BiTE fragments at the concentration corresponding to saturation concentration for staining, in RPMI with 10% FCS and 2 mM L-glutamine, sodium pyruvate, 100 U/mL penicillin, and 100 μg/mL streptomycin, at 200,000 cells/well, on ice and at 37 °C for 2 h, in triplicates. Antibody fragments were diluted to 300 nM (omalizumab), 35 nM (8D6), 10 nM (ligelizumab and MEDI4212), 200 nM (quilizumab), and 20 nM (blinatumomab). The samples were then placed on ice for 5 min, resuspended in 10 µg/mL anti-FLAG antibody (Sigma-Aldrich, St. Louis, MO, USA) in 2% BSA-PBS, and incubated on ice for 30 min. Binding of anti-FLAG was detected with goat anti-mouse-FITC conjugate (Sigma-Aldrich, St. Louis, MO, USA), diluted 1:200 in 2% BSA-PBS after a 30-min-incubation on ice. The cells were resuspended in 200 µL ice-cold PBS and analyzed with Guava^®^ EasyCyte™ Flow Cytometer (Luminex, Austin, TX, USA).

### 2.9. T-Cell Activation Assay

A reporter assay based on CD3-overexpressing cells transformed with a vector that enables luciferase activity following the activation of nuclear factor of activated T cells (NFAT) promoter element (Promega, Madison, WI, USA) was used to assess the level of activation of T cells upon the contact with the target cells covered with a bispecific antibody. The target cell line and the control cell line transformed with an empty vector were induced with doxycycline for 48 h. Fcε-expressing cells and control empty-vector-transformed cells were distributed in aliquots of 20,000 cells with an effector to target (E:T) ratio of 5:1, or 100,000 cells for an E:T ratio 1:1, to the wells of a 96-well-cell culture plate in 25 µL. A total of 25 µL of BiTE-antibodies were added in a 5-fold dilution series, starting at 1 nm for blinatumomab, 10 nM for omalizumab-, 8D6-, ligelizumab-, and MEDI4212-, and 75 nm for quilizumab-based-BiTE. A total of 25 µL aliquots of the effector cells (approximately 100,000 cells/well) were delivered.

Another assay using CD3-overexpressing cells where luciferase activity can be detected following the activation of interleukin-2 (IL-2) promoter element (Promega, Madison, WI, USA) was performed in a similar manner using an E:T ratio of 2.5.

All measurements were performed at least in duplicates. After a 5-h-incubation at 37 °C, under 5% CO_2_ in a hydrated atmosphere, an equal volume of luminescence substrate was added to the wells, and the signals were determined using a Sunrise™ spectrophotometer (Tecan, Männedorf, Switzerland). Data in relative luminescence units (RLU) was processed using the equation “fold induction” = RLU (induced–background)/RLU (no antibody control–background).

### 2.10. Upregulation of the Activation Markers CD69 and CD25 on Human T Cells

Human CD8-positive cells were isolated using a RosetteSep Human CD8^+^ T cell enrichment cocktail (STEMCELL Technologies, Vancouver, BC, Canada). A total of 50 µL of the reagent were added per 2 mL whole blood and incubated for 20 min at RT. The sample was then diluted with an equal volume of 2% FCS-PBS and gently mixed, and then layered on top of the Lymphoprep density gradient medium (STEMCELL Technologies, Vancouver, BC, Canada) in SepMate-50 tubes (STEMCELL Technologies, Vancouver, BC, Canada). After centrifugation for 10 min at 1200× *g* at room temperature, enriched cells in the top layer were removed and washed twice with 2% FCS-PBS at 300× *g* for 8 min at RT. For the activation experiment, 30,000 effector cells were co-cultured in RPMI-1640 with 10% FCS, 2 mM L-glutamine, sodium pyruvate, 100 U/mL penicillin, and 100 μg/mL streptomycin with induced Ramos-Fcε cells or Ramos cells, transformed with vector only, at an E:T ratio of 1:1 per well of a 96-U-shaped well plate. The response to 1 nM blinatumomab, 2 nM 8D6-BiTE, and 2 nM ligelizumab-BiTE was measured, and wells with no antibody were included as controls. All set-ups were prepared in duplicates.

After a 48-h-incubation at 37 °C in a humidified atmosphere under 5% CO_2_, cells were resuspended in 2% BSA-PBS and stained with anti-CD8-APC antibody (clone RPA-T8, STEMCELL Technologies, Vancouver, BC, Canada) at a 1:50 dilution. For the detection of activation markers, the conjugates against CD69 (PE-labeled anti-human CD69 mAb (#FN50, Biolegend, San Diego, CA, USA)) at 1:100 dilution, CD25 (PE-labeled anti-human CD25 mAb clone BC96, BioLegend, San Diego, CA, USA) at 1:20 dilution, and PE-labelled isotype control (clone MOPC21, BD Biosciences, Franklin Lakes, NJ, USA) at 1:300 dilution were used. After incubation for 30 min on ice, the cells were collected with centrifugation at 300× *g* for 5 min at 4 °C, resuspended in ice-cold PBS, and analyzed using a Guava^®^ EasyCyte™ Flow Cytometer (Luminex, Austin, TX, USA).

## 3. Results

### 3.1. Expression and Purification of Bispecific Antibodies

**Figure 1:**
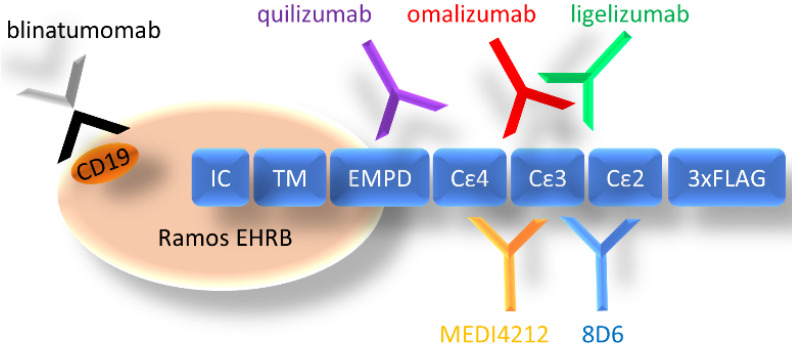
Organization of cell-expressed IgE-Fc and schematic depiction of the location of epitopes of targeting antibodies (CD19 for blinatumomab). IC: intracellular domain, TM: transmembrane domain, EMPD: extracellular membrane–proximal domain, Cε2–4: Fcε domains 2–4, 3× FLAG: 3 repeats of FLAG-tag.

**Figure 2:**
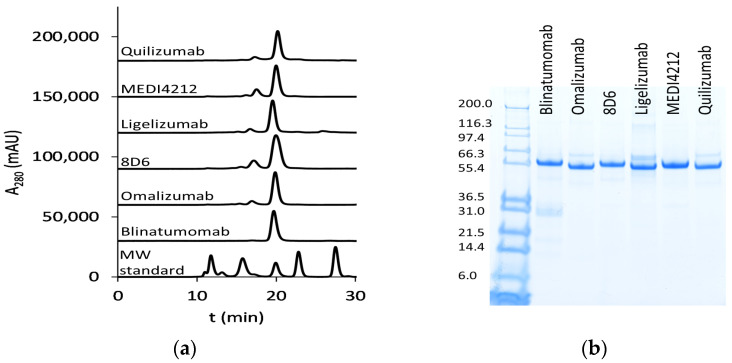
Purified bispecific BiTE antibodies: (**a**) HPLC-SEC profiles of purified bispecific BiTE antibodies. MW (molecular weight) standard (Bio-RAD) includes thyroglobulin (670 kDa), bovine γ-globulin (158 kDa), chicken ovalbumin (44 kDa), equine myoglobin (17 kDa), and vitamin B12 (1.3 kDa); (**b**) SDS-PAGE analysis (first lane: Mark12 unstained standard (Thermo Fisher Scientific, Waltham, MA, USA) with MW in kDa).

We have chosen a panel of Fcε targeting antibodies with different epitopes (Figure 1) as donors of variable regions in bispecific fusion constructs of two single-chain antibodies where one unit interacts with CD3ε, potentially enabling potent activation of T-cells. Using an ExpiCHO expression system and a single-step IMAC purification, the molecules were isolated at 17.7–71.5 mg/L culture (Supplementary Table S3). In HPLC-SEC, in native conditions, the bispecific antibodies eluted at the time indicated their expected molecular weight at about 55 kDa (Figure 2a), as was also observed on SDS-PAGE (Figure 2b).

### 3.2. Cell Surface Staining

**Figure 3:**
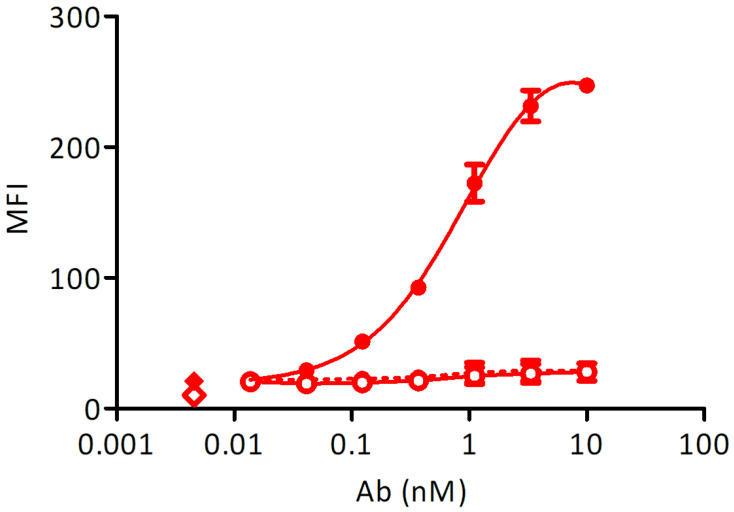
Test of Fcε expression using staining with Xolair^®^: Full circles-full line: doxycycline-induced Ramos-Fcε cell line, empty circles-full line: doxycycline-induced Ramos cell line transformed with an empty vector, full circles-dashed line: uninduced Ramos-Fcε cell line, full diamond: cells stained with secondary reagent only, empty diamond: cells only. All measurements were done at least in duplicates. MFI: mean fluorescence units resulting from bound secondary reagent anti-human-IgG-phycoerythrin (PE).

To control the expression of membrane-bound Fcε, a stable cell line transformed with an inducible construct was measured at a peak expression of Fcε after a 48 h induction with 1 µg/mL doxycycline. The cell line, transformed with an empty vector and induced at the same conditions and non-induced cells were used as controls. Induced Ramos EHRB-Fcε cells could bind the control antibody with an EC_50_ of 1.0 nM, while no reactivity with Xolair^®^ could be detected in the absence of doxycycline, and also the staining of a cell line transformed with an empty construct was in the background range (Figure 3).

**Figure 4:**
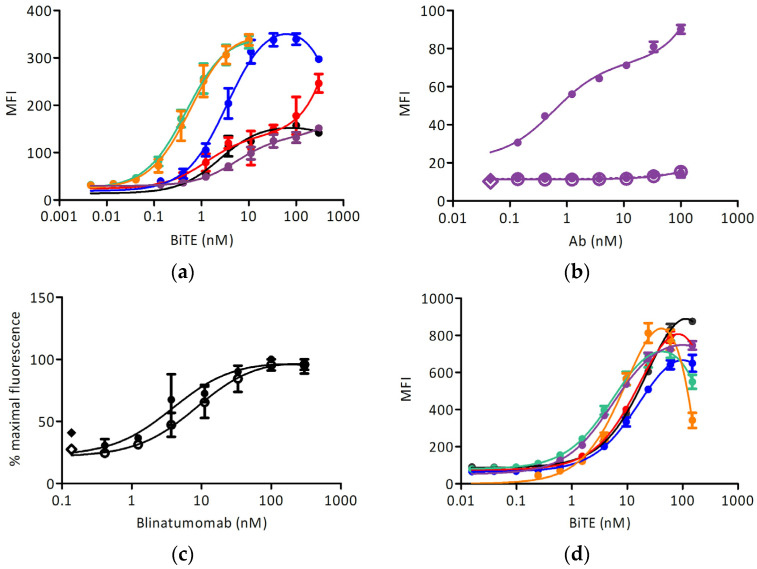
Cell surface staining experiments: (**a**) Binding of BiTE antibodies to the Ramos-Fcε cell line (black: blinatumomab, red: omalizumab, blue: 8D6, green: ligelizumab, orange: MEDI4212, purple: quilizumab); (**b**) Binding of quilizumab to Ramos-Fcε cell line (full circles-full line: induced Ramos-Fcε cell line, empty circles-full line: doxycycline-induced Ramos cell line transformed with an empty vector, full circles-dashed line: uninduced Ramos-Fcε cell line, full diamond: cells stained with secondary reagent only, empty diamond: cells only); (**c**) Binding of blinatumomab to: induced Ramos-Fcε cell line (full circles-full line) and induced Ramos cell line transformed with an empty vector (empty circles-full line). Full diamond: cells stained with secondary reagent only, empty diamond: cells only; (**d**) Binding of BiTE antibodies to the Jurkat E6-1 cell line (color scheme as in (**a**)). All measurements were done at least in duplicates. MFI: mean fluorescence units resulting from the signal of bound antibody conjugates (**a**,**c**) anti-pentahis-AlexaFluor^®^ 488, (**b**) anti-human-IgG-phycoerythrin (PE), (**d**) anti-pentahis-AlexaFluor^®^ 647.

Different anti-IgE BiTEs stained the induced target cell line with different EC_50_ (Figure 4a and Table 1); no reactivity with Ramos-EHRB was measured for any IgE-targeting antibody tested. The signals obtained with the quilizumab-based BiTE were lower than for other antibodies, and so we examined the binding of full-length quilizumab-IgG (Figure 4b). Furthermore, the signal was much lower than upon staining with omalizumab-IgG (Figure 3). The binding of blinatumomab was in the same range for Fcε-expressing cells, and the control cell line and an EC_50_ of about 5 nM could be determined (Figure 4c). Further, the reactivity with CD3-positive Jurkat 6E-1 cells was measured (Figure 4d): here, all BiTE-constructs could react with this cell line at a similar extent with an EC_50_ of about 20 nM. No binding to CD3-negative cell line TIB-153 was detected.

### 3.3. Immunofluorescence Microscopy

**Figure 5:**
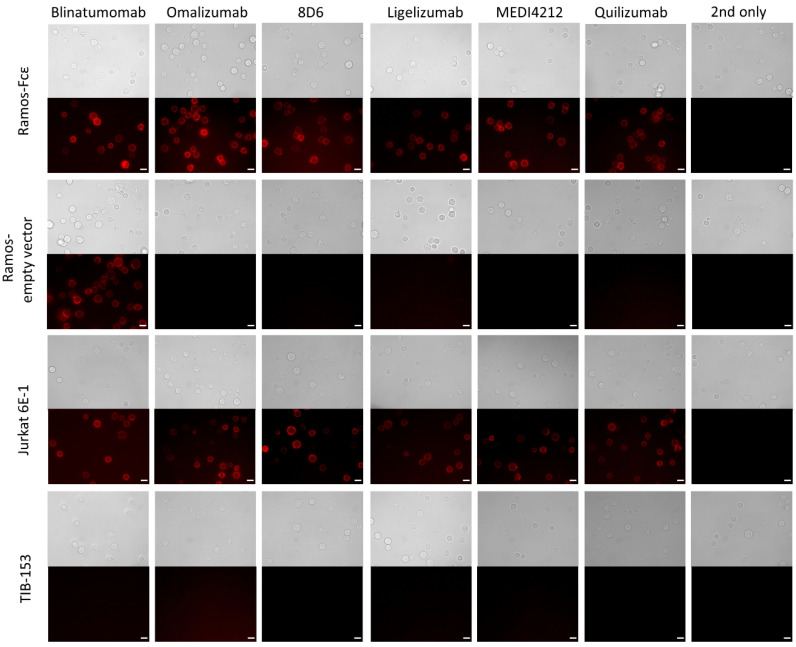
Immunofluorescence microscopy to observe the binding to cell-bound targets of bispecific antibodies: Binding of BiTE antibodies to the Ramos-Fcε cell line; the Ramos cell line transformed with the empty vector, CD3-positive Jurkat E6-1 cells, and CD3-negative TIB-153 cells. Cells stained with secondary reagent only (2nd only) were used as a negative control. Scale bar represents 10 µm.

We then used immunofluorescence to examine cell surface binding of the constructs to the Ramos-Fcε cell line; the Ramos cell line transformed with the empty vector, CD3-positive Jurkat E6-1 cells, and CD3-negative TIB-153 cells. Blinatumomab stained both types of Ramos cells and other BiTE-constructs stained only the Fcε-positive cell line. All constructs stained Jurkat cells while no staining of TIB-153 cells could be observed (Figure 5).

### 3.4. Internalization of Fcε after Incubation with BiTE Fragments

**Figure 6:**
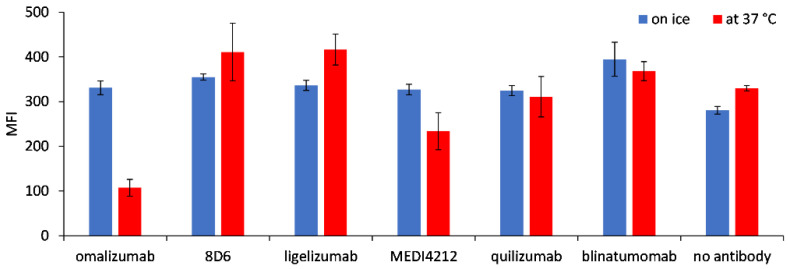
Internalization of Fcε, expressed on Ramos-EHRB cells, upon incubation with BiTE antibodies. Mean fluorescence intensity (MFI) values resulting from binding of the anti-FLAG antibody detected with an anti-mouse-FITC conjugate are shown for the cells treated with BiTEs on ice (blue bars) or 37 °C (red bars), in triplicate measurements (mean ± S.D.).

We tested the possible internalization of the target molecule Fcε into Ramos-EHRB cells as a consequence of incubation with BiTE fragments by comparing the binding of anti-FLAG antibodies when cells were treated at 37 °C or on ice. While the reactivity was similar for the cells incubated in both tested conditions with 8D6, ligelizumab, and quilizumab-based BiTEs, as well as for blinatumomab, a reduction in fluorescence level was measured for 37 °C-incubated cells by 67.5% for omalizumab and 20.5% for MEDI4241-based BiTE, compared with those treated on ice (Figure 6).

### 3.5. T-Cell Activation Assays

**Figure 7:**
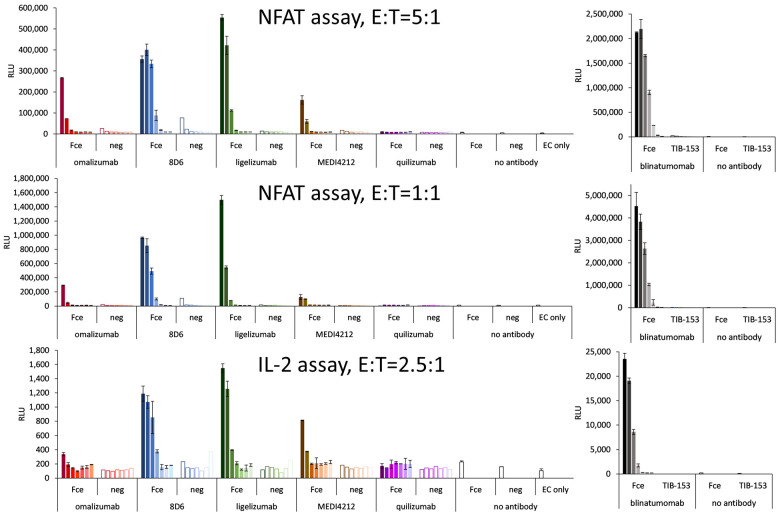
T-cell activation assays with Ramos-Fcε—targeting BiTE-like fragments: T-cell activation as a response to IgE-targeting BiTE-fragments using graded concentrations of BiTE starting with 400 pM for omalizumab-, MEDI4212-, and quilizumab-based molecules, and 2 nM for 8D6- and ligelizumab-based BiTEs (full bars: Fcε-cell line, empty bars: control cell line) and T-cell activation caused by dilutions of blinatumomab starting at 1 nM, when bound to Ramos-Fcε cells with the response of TIB-153 as a negative cell line. NFAT: nuclear factor of activated T cells, IL-2: interleukin 2, E:T: effector to target cell ratio.

Bispecific constructs were tested for the specific activation of T cells in a reporter cell assay based on engineered CD3-overexpressing T cells at an E:T ratio of 5:1. Specific activation could be demonstrated for 8D6 and ligelizumab-based BiTEs (Figure 7). The level of activation was lower than for blinatumomab, which could activate the T cells with an EC_50_ of 14 pM (Figure 7), nevertheless in the case of 8D6 BiTE, a 37-fold specific induction could be observed at an 80 pM concentration, where the response to the target cells with no Fcε expression did not exceed the background. The ligelizumab BiTE caused a 47-fold induction at 400 pM with no activation using Fcε-negative cells. The omalizumab BiTe caused an 11-fold specific induction as opposed to a 2-fold response of the Fcε-negative cells at a concentration of 80 pM. The MEDI4212 BiTE was similar, with a 9-fold specific induction vs. a 2-fold for the negative cells at 80 pM. No response for any of the cell lines was measured for quilizumab BiTE.

At an E:T ratio of 1:1, 8D6 BiTE induced a 53-fold specific activation with no response to the control cell line at 80 pM, and the response to ligelizumab BiTE was 162-fold at 2000 pM and 59-fold at 400 pM. The effect of Omalizumab BiTE was 5-fold activation at 80 pM, and of MEDI4212 BiTE was 10-fold at 400 pM. Again, quilizumab BiTE did not induce any response. Importantly, for 8D6 and ligelizumab BiTE, the effect on the negative cells was similar as when assayed at an E:T ratio of 1:1, and only the fold change of the effect on the positive cell line was increased.

In the activation test of T cells employing an IL-2 promoter element as the driver of luciferase expression, 8D6 BiTE caused a six-fold and ligelizumab BiTE caused a seven-fold specific activation at 2 nM, omalizumab, and MEDI4212 BiTEs were less potent with two- and four-fold activation at 400 pM, and there was no measurable response for quilizumab BiTE.

### 3.6. Expression of Activation Markers of CD8^+^ T Cells

**Figure 8:**
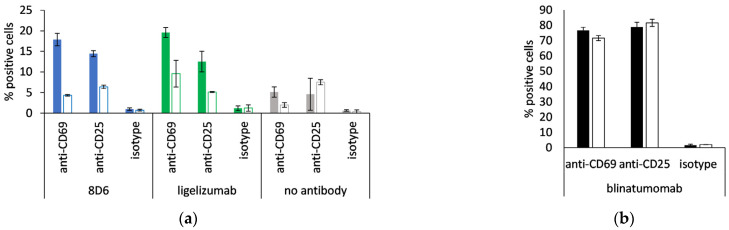
Activation of CD8-positive T cells. Percent CD69-positive and CD25-positive cytotoxic T cells when incubated with (**a**) 8D6- and ligelizumab-based BiTE or without antibody; (**b**) blinatumomab. Full bars: Ramos-Fcε cells, empty bars: Ramos cells, transformed with empty vector; duplicate measurements are presented (mean ± S.D.).

8D6- and ligelizumab-based BiTEs were tested for the ability of activation of non-stimulated CD8-positive cells, monitored via the expression of CD69 and CD25 at an E:T ratio of 1:1. When the effector cells were incubated with Fcε-expressing Ramos cells, the percentage of positive cells for each activation marker was two- to four-fold, compared with when they were incubated with the Ramos cell line, transformed with the empty vector, or when no antibody was present (Figure 8a). Blinatumomab was used as a positive control, and 70–80% of the CD8-positive cells were measured to be CD69- or CD25-positive after a co-culturing with the above cell lines, both of which are CD19-positive (Figure 8b).

## 4. Discussion

As the interaction with polyvalent antigen, followed by crosslinking of FcεRI, leads to the strong activation of mast cells and basophils, this molecule as an IgE ligand is considered a target for the development of biologics for the treatment of allergic disease, including atopic dermatitis, seasonal rhinitis, urticaria, life-threatening anaphylaxis, and prolonged inflammation leading to chronic conditions, such as asthma. These therapeutics include monoclonal antibody approaches, as well as treatment with multispecific reagents, including DARPins [27,28] and combination approaches [29], and their modes of action extend from binding soluble IgE over inhibition to binding of IgE to FcεRI, a decrease of FcεRI expression, to the blockade of FcεRI signaling, including FcγRIIb-FcεRI coaggregation [29,30,31,32,33]. Significant and possible longer sustained reduction of serum IgE could be achieved by lysis of the membrane IgE-expressing B lymphoblasts and consequent prevention of the generation of IgE-producing plasma cells. Apoptosis of BCR-expressing cells upon the receptor crosslinking has been suggested as an effective means of reducing IgE B cell numbers and was observed with polyclonal [34] and monoclonal reagents targeting the EMPD domain [26] or the Cε3 domain of the membrane-bound IgE. ADCC-mediating antibodies, whose activity could be enhanced with mutations or modifications in glycosylation pattern causing a more potent interaction with effector cells [18], as well as the application of CTLs against IgE-producing B cells [10], have already proven efficient in inhibiting IgE responses to antigenic challenges for a long period, suggesting that the elimination of IgE-producing B cells and plasma cells by IgE-specific CTLs could be a more efficient approach to the treatment of allergic disorders.

Here we expressed five bispecific antibodies in a format of two single-chain Fv fragments in a single polypeptide chain, each featuring an anti-CD3ε subunit that can mediate activation of T-cells and different Fv fragments originating from anti-IgE antibodies reacting with various IgE epitopes. All planned constructs could be produced in a mammalian cell expression system at a small scale. The specific reactivity with cells expressing membrane-bound IgE-Fc was demonstrated for all constructs expressed in this format, using flow cytometry and immunofluorescence microscopy; a quilizumab-based BiTE fragment showed only a low level of staining compared with other constructs, but also full-length quilizumab IgG was less reactive than the Xolair^®^ antibody used as a control. Partly, this can correlate with different antigen affinities, but also with different accessibility of the cognate epitope, located in the EMPD of the Fcε. We also observed internalization of Fcε after incubation with omalizumab, and to a lesser extent, with a MEDI4212-based construct, which could have contributed to their low activity in the T-cell activation assay. All expressed BiTE-like fragments could specifically stain CD3-positive Jurkat cells at a similar level. In the T-cell activation assay, 8D6- and ligelizumab-based BiTEs clearly induced strong specific activation of T cells when in contact with the Fcε-expressing cell line, but the level of activation was much lower than what was measured for blinatumomab. Both BiTE fragments induced the expression of activation markers on non-stimulated CD8-positive cells. Regarding that, with BiTE-like fragments, the engagement of the epitope on the B-cell is only monovalent; optimization of the affinity for monovalent Fcε appears the next logical step in the development of CTL killing-mediating constructs.

Through the study of isolated protein domains that act as a target molecule for BiTEs, it has been demonstrated that cells expressing small target antigens are generally better lysed than those expressing large complex target antigens, as they are easily accommodated in the synaptic cleft at large copy numbers [35]. At the same time, the antigen size also determines the size of the intracellular bridge formed by the target, BiTE construct, and CD3ε subunit, which is optimally considered to be 14 nm, corresponding to the length of a natural TCR/peptide/MHC complex [35], and after subtracting 3.3 nm of a typical BiTE and 4 nm of the CD3ε subunit, the remaining 7.7 nm could just about fit the optimal distance from the cell surface to the binding sites of the tested anti-IgE antibodies (which is difficult to judge more precisely because the EMPD is intrinsically unstructured [26]), and the effect of anti-IgE BiTEs could, in the future, be optimized by testing a larger panel of antibodies binding to different epitopes of membrane-expressed IgE, to find one that more efficiently contributes to the formation of immune synapse and can hence elicit a more potent T-cell activation. Further, as serum levels of soluble IgE rises substantially in allergic patients, anti-IgE antibodies that can bind to cell-bound IgE but are not reactive with soluble IgE could be of advantage as donors of variable domains in CTL killing-mediating constructs. Specific obliteration of IgE B lymphocytes, the prime source of IgE, with a strategy employing membrane-IgE targeting BiTEs would, at the same time, not deleteriously affect other B-cell populations and avoid the adverse effects resulting from such therapies [36].

## Figures and Tables

**Table 1:** EC_50_ of binding of BiTE-like constructs to the cell line Ramos-Fcε (mean and S.D.).

BiTE Fragment	EC_50_ for Binding to Ramos-Fcε Cells (nM)
Blinatumomab	2.5 ± 0
Omalizumab	2.0 ± 0.8
8D6	2.3 ± 1.3
Ligelizumab	0.4 ± 0.04
MEDI4212	0.4 ± 0.2
Quilizumab	~40

## Data Availability

The data presented in this study are available within this article or its supplementary material.

## Supplementary Materials

The following are available online at https://www.mdpi.com/article/10.3390/biomedicines9111568/s1, Table S1: Amino acid sequences of antibody constructs, Table S2: Oligonucleotide sequences for PCR amplification of BiTE-encoding fragments, Table S3: Expression yields of BiTE-like constructs.
