# Supplementary material for: Bispecific T-Cell Engagers Targeting Membrane-Bound IgE
Source: Biomedicines. 2021 Oct 29;9(11):1568. doi: 10.3390/biomedicines9111568 (PMC8615095; doi:10.3390/biomedicines9111568)
Supplement: Supplementary file 1 [file biomedicines-09-01568-s001.zip › SupplementaryTable S1.pdf]

Supplementary Table S1. Amino acid sequences of antibody constructs

(Anti-CD3ε in gray and anti-IgE variable domains in black (blinatumomab), red (omalizumab), blue (8D6), green (ligelizumab), orange (MEDI 4212) and purple (quilizumab). Short linker is underlined with full line and long linker with dotted line)

Amino acid sequences of antibody constructs

Blinatumomab

DIQLTQSPASLAVSLGQRATISCKASQSVDYDGDSYLNWYQQIPGQPPKLLIYDASNLVSGIPPRFSGSGSGTDFTLNI
HPVEKVDAATYHCQQSTEDPWTFGGGTKLEIKGGGGSGGGGSGGGGSQVQLQQSGAELVRPGSSVKISCKASGYA
FSSYWMNWVKQRPGQGLEWIGQIWPGDGDTNYNGKFKGKATLTADESSSTAYMQLSSLASEDSAVYFCARRETT
TVGRYYYAMDYWGQGTTVTVSSGGGGSDIKLQQSGAELARPGASVKMSCKTSGYTFTRYTMHWVKQRPGQGLE
WIGYINPSRGYTNYNQKFKDKATLTTDKSSSTAYMQLSSLTSEDSAVYYCARYYDDHYCLDYWGQGTTLTVSSVEGG
SGGSGGSGGSGGVDDIQLTQSPAIMSASPGEKVTMTCRASSSVSYMNWYQQKSGTSPKRWIYDTSKVASGVPYRF
SGSGSGTSYSLTISSMEAEDAATYYCQQWSSNPLTFGAGTKLELKGSSTG**HHHHHHHHG**

Omalizumab BiTE

DIQLTQSPSSLSASVGDRVTITCRASQSVDYDGDSYMNWYQQKPGKAPKLLIYAASYLESGVPSRFSGSGSGTDFTLTI
SSLQPEDFATYYCQQSHEDPYTFGCGTKVEIKGGGGSGGGGSGGGGSEVQLVESGGGLVQPGGSLRLSCAVSGYSIT
SGYSWNWIRQAPGKCLEWVASITYDGSTNYNPSVKGRITISRDDSKNTFYLQMNSLRAEDTAVYYCARGSHYFGH
WHFAVWGQGTLVTVSSGGGGSDIKLQQSGAELARPGASVKMSCKTSGYTFTRYTMHWVKQRPGQGLEWIGYIN
PSRGYTNYNQKFKDKATLTTDKSSSTAYMQLSSLTSEDSAVYYCARYYDDHYCLDYWGQGTTLTVSSVEGGSGGSG
GSGGSGGVDDIQLTQSPAIMSASPGEKVTMTCRASSSVSYMNWYQQKSGTSPKRWIYDTSKVASGVPYRFSGSGS
GTSYSLTISSMEAEDAATYYCQQWSSNPLTFGAGTKLELKGSSTG**HHHHHHHHG**

8D6 BiTE

DIVLTQSPASLAVSLGQRATISCKASQSVDYDGDTYMNWYHQKPGQPPKLLIYAASNLDSGIPARFSGSGSGTDFTL
NIHPVEEEDAATYYCQQTNEDPWTFGGGTKLEIKGGGGSGGGGSGGGGSQVQLQQSGAELAKPGASVMLSCKAS
GYTFNGYWMHWVKQRPGQDLEWIGYINPTTGHTEYNQKFKDKATLTADESSNTAYIELSSLTSDDSAVYYCARQEY
RHSWFAYWGQGTLVTVSAGGGGSDIKLQQSGAELARPGASVKMSCKTSGYTFTRYTMHWVKQRPGQGLEWIGYI
NPSRGYTNYNQKFKDKATLTTDKSSSTAYMQLSSLTSEDSAVYYCARYYDDHYCLDYWGQGTTLTVSSVEGGSGGS
GGSGGSGGVDDIQLTQSPAIMSASPGEKVTMTCRASSSVSYMNWYQQKSGTSPKRWIYDTSKVASGVPYRFSGSG
SGTSYSLTISSMEAEDAATYYCQQWSSNPLTFGAGTKLELKGSSTG**HHHHHHHHG**

Ligelizumab BiTE

EIVMTQSPATLSVSPGERATLSCRASQSIGTNIHWYQQKPGQAPRLLIYYASESISGIPARFSGSGSGTEFTLTISSLQSE
DFAVYYCQQSWSWPTTFGGGTKVEIKGGGGSGGGGSGGGGSQVQLVQSGAEVMKPGSSVKVSCKASGYTFSWY
WLEWVRQAPGHGLEWMGEIDPGTFTTNYNEKFKARVTFTADTSTSTAYMELSSLRSEDTAVYYCARFSHFSGSNYD
YFDYWGQGTLVTVSSGGGGSDIKLQQSGAELARPGASVKMSCKTSGYTFTRYTMHWVKQRPGQGLEWIGYINPSR
GYTNYNQKFKDKATLTTDKSSSTAYMQLSSLTSEDSAVYYCARYYDDHYCLDYWGQGTTLTVSSVEGGSGGSGGSG
GSGGVDDIQLTQSPAIMSASPGEKVTMTCRASSSVSYMNWYQQKSGTSPKRWIYDTSKVASGVPYRFSGSGSGTSY
SLTISSMEAEDAATYYCQQWSSNPLTFGAGTKLELKGSSTG**HHHHHHHHG**

MEDI4212 BiTE

QSVLTQPPSVSGAPGQRVTISCTGSSSNIGAGYDVHWYQQLPGTAPKLLIYDNFNRPSGVPDRFSGSKSGTSASLAIT
GLQAEDEADYYCQSYDSPTLTSPFGTGTKLTVLGGGGSGGGGSGGGGSEVQLVQSGAEVKKPGATVKISCKVYGYIF
TDYNIYWVRQAPGKGLEWMGLIDPDNGETFYAEKFQGRATMTADTSSDRAYMELSSLRFEDTAVYYCATVMGKW
IKGGYDYWGRGTLVTVSSGGGGSDIKLQQSGAELARPGASVKMSCKTSGYTFTRYTMHWVKQRPGQGLEWIGYIN
PSRGYTNYNQKFKDKATLTTDKSSSTAYMQLSSLTSEDSAVYYCARYYDDHYCLDYWGQGTTLTVSSVEGGSGGSG
GSGGSGGVDDIQLTQSPAIMSASPGEKVTMTCRASSSVSYMNWYQQKSGTSPKRWIYDTSKVASGVPYRFSGSGS
GTSYSLTISSMEAEDAATYYCQQWSSNPLTFGAGTKLELKGSSTG**HHHHHHHHG**

Quilizumab BiTE

DIQMTQSPSSLSASVGDRVTITCRSSQSLVHNNANTYLHWYQQKPGKAPKLLIYKVSNRFSGVPSRFSGSGSGTDFTL
TISSLQPEDFATYYCSQNTLVPWTFGQGTKVEIKGGGGSGGGGSGGGGSEVQLVESGGGLVQPGGSLRLSCAASGF
TFSDYGIAWVRQAPGKGLEWVAFISDLAYTIYYADTVTGRFTISRDNSKNTLYLQMNSLRAEDTAVYYCARDNWDA
MDYWGQGTLVTVSSGGGGSDIKLQQSGAELARPGASVKMSCKTSGYTFTRYTMHWVKQRPGQGLEWIGYINPSR
GYTNYNQKFKDKATLTTDKSSSTAYMQLSSLTSEDSAVYYCARYYDDHYCLDYWGQGTTLTVSSVEGGSGGSGGSG
GSGGVDDIQLTQSPAIMSASPGEKVTMTCRASSSVSYMNWYQQKSGTSPKRWIYDTSKVASGVPYRFSGSGSGTSY
SLTISSMEAEDAATYYCQQWSSNPLTFGAGTKLELKGSSTG**HHHHHHHHG**

Quilizumab – full length IgG

Heavy chain:

EVQLVESGGGLVQPGGSLRLSCAASGFTFSDYGIAWVRQAPGKGLEWVAFISDLAYTIYYADTVTGRFTISRDNSKNT
LYLQMNSLRAEDTAVYYCARDNWDAMDYWGQGTLVTVSSASTKGPSVFPLAPSSKSTSGGTAALGCLVKDYFPEP
VTVSWNSGALTSGVHTFPAVLQSSGLYSLSSVVTVPSSSLGTQTYICNVNHKPSNTKVDKKVEPKSCDKTHTCPPCPA
PELLGGPSVFLFPPKPKDTLMISRTPEVTCVVVDVSHEDPEVKFNWYVDGVEVHNAKTKPREEQYNSTYRVVSVLTV
LHQDWLNGKEYKCKVSNKALPAPIEKTISKAKGQPREPQVYTLPPSRDELTKNQVSLTCLVKGFYPSDIAVEWESNG
QPENNYKTTPPVLDSDGSFFLYSKLTVDKSRWQQGNVFSCSVMHEALHNHYTQKSLSLSPGK

Light Chain:

DIQMTQSPSSLSASVGDRVTITCRSSQSLVHNNANTYLHWYQQKPGKAPKLLIYKVSNRFSGVPSRFSGSGSGTDFTL
TISSLQPEDFATYYCSQNTLVPWTFGQGTKVEIKRTVAAPSVFIFPPSDEQLKSGTASVVCLLNNFYPREAKVQWKVD
NALQSGNSQESVTEQDSKDSTYSLSSTLTLSKADYEKHKVYACEVTHQGLSSPVTKSFNRGEC
