# Supplementary material for: Bispecific T-Cell Engagers Targeting Membrane-Bound IgE
Source: Biomedicines. 2021 Oct 29;9(11):1568. doi: 10.3390/biomedicines9111568 (PMC8615095; doi:10.3390/biomedicines9111568)
Supplement: Supplementary file 1 [file biomedicines-09-01568-s001.zip › SupplementaryTable S2.pdf]

Supplementary Table S2. Oligonucleotide sequences for PCR amplification of BiTE-encoding fragments.

BiTE construct/Oligonucleotide	Nucleotide sequence (restriction sites underlined)
Blinatumomab	
Bl_bite_nhe1	acgtgctagc gacatccagc tgacac
Bl_bite_bam2	attgggatcc cttcagctcc agcttg
Omalizumab	
Oma_bite_nhe1	acgtgctagc gacatccagc tgacccag
Oma_bite_ecorv2	acgtgatatc gcttccgccg ccaccgctgc tcacggtcac cag
8D6	
8D6_bite_nhe1	acgtgctagc gacatcgtgc tgacccag
8D6_bite_ecorv2	acgtgatatc gcttccgccg ccaccggcgc tcacggtcac cag
Ligelizumab	
Lig_bite_nhe1	tgcagctagc gagatcgtga tgacccag
Oma_bite_ecorv2	acgtgatatc gcttccgccg ccaccgctgc tcacggtcac cag
MEDI4212	
Medi_bite_nhe1	acgtgctagc cagagcgtgc tgacccag
Oma_bite_ecorv2	acgtgatatc gcttccgccg ccaccgctgc tcacggtcac cag
Quilizumab	
Qui_bite_nhe1	acgtgctagc gatattcaga tgactcag
Qui_bite_ecorv2	acgtgatatc gcttccgccg ccaccgctgg acactgtcac cag
