# Supplementary material for: Bispecific T-Cell Engagers Targeting Membrane-Bound IgE
Source: Biomedicines. 2021 Oct 29;9(11):1568. doi: 10.3390/biomedicines9111568 (PMC8615095; doi:10.3390/biomedicines9111568)
Supplement: Supplementary file 1 [file biomedicines-09-01568-s001.zip › SupplementaryTable S3.pdf]

Supplementary Table S3. Expression yields of BiTE-like constructs.

BiTE fragment	Yield after IMAC purification per L culture (mg)
Blinatumomab	47.6
Omalizumab	39.9
8D6	45.7
Ligelizumab	71.5
MEDI4212	56.8
Quilizumab	17.7
